# Description of *Hypogastrura ellisi* sp. n. with notes on *H. tethyca* Ellis and the *trybomi* group (Collembola, Hypogastruridae)

**DOI:** 10.3897/zookeys.719.14806

**Published:** 2017-12-07

**Authors:** Dariusz Skarżyński, Morteza Kahrarian, Agata Piwnik, Marlena Zawisza

**Affiliations:** 1 Institute of Environmental Biology, University of Wrocław, Przybyszewskiego 65, 51-148 Wrocław, Poland; 2 Young researchers and elite club, Kermanshah branch, Islamic Azad University, Kermanshah, Iran

**Keywords:** Azerbaijan, Greece, Iran, key, springtails, taxonomy

## Abstract

A new species, *Hypogastrura ellisi*, from Iran and Greece is described. It does not fit the definition of any known species group, but, as it has weakly differentiated blunt Ant. IV sensilla, one tenent hair on tibiotarsi, broad empodial lamellae, and dens with fine granulation and seven setae, it can be compared with some members of the *trybomi* and *monticola* groups and *H. aterrima* Yosii, 1972. It seems to be especially close to a representative of the *trybomi* group, *H. tethyca* Ellis, 1976. They differ mainly in the shape of apical papillae on the labrum, the size of anal spines, and the presence of setae m_2_ on Th. II. Notes on *H. tethyca*, the *trybomi* group, and a key to the species of the group are given.

## Introduction

*Hypogastrura* Bourlet, 1839 currently comprising 168 species (Bellinger et al. 1996‒2017), is the most speciose genus within the family Hypogastruridae. Looking for phylogenetic relationships between them, and for practical reasons, the genus has been divided into some species groups based on morphology (Yosii 1960, Christiansen and Bellinger 1980, Babenko et al. 1994, Skarżyński 2009). Presently, nine groups are used in the taxonomy of the genus: *crassaegranulata*, *manubrialis*, *monticola*, *nivicola*, *packardi, sahlbergi*, *socialis*, *trybomi*, and *viatica*. This group system is not complete; numerous species do not belong to any of these groups due to their specific morphology or poor knowledge on their morphology. In the material collected in Iran, several specimens that resemble *Hypogastrura tethyca* Ellis, 1976, a member of the *trybomi* group, were found. Studies on the type material of this species made it possible to ascertain that the Iranian specimens represented a new species that does not fit the definition of any known species group. Its description, notes on *H. tethyca* and the *trybomi* group as a whole, as well as a key to all known species of the group are given below.

## Materials and methods

Specimens of *Hypogastrura ellisi* sp. n. were cleared in Nesbitt’s fluid (Wang et al. 2003), subsequently mounted on slides in Swan’s medium (Swan 1936) and studied using a Nikon Eclipse E600 phase contrast microscope. Figures were drawn with the camera lucida. Photographs were made using a camera Nikon D5100 mounted on a microscope mentioned above. Photographs were stacked using Helicon Focus 6.7.1. and prepared for publication using Adobe Photoshop CS6.

Terminology for the description follows that given in Fjellberg (1984, 1999), Babenko et al. (1994) and Thibaud et al. (2004).

Abbreviations used:

**Ant. I–IV** antennal segments I–IV,

**Th. I–III** thoracic terga I–III,

**Abd. I–VI** abdominal terga I–VI.

## Taxonomy

### Hypogastrura ellisi

Taxon classificationAnimaliaCollembolaHypogastruridae

Skarżyński & Kahrarian sp. n.

http://zoobank.org/C8F33D89-AEDF-45FB-B748-2078A73A5F2B

Figs 1–3 , 4–8 , 9–10

#### Type material.

Holotype: female on slide, litter in oak forest, Zagros Mountains, Dalab mountain (33°34'N, 47°31'E / 1700 m a.s.l.), Kohdasht County, Lorestan Province, Iran, 4.XII.2013, leg. M. Kahrarian. Paratypes: 4 females, 1 male, same data as holotype; 1 female, 1 male, litter in oak forest, Zagros Mountains, Sorkhdom mountain (33°34'N, 47°32'E / 1650 m a.s.l.), Kohdasht County, Lorestan Province, Iran, 14.XI.2013, leg. M. Kahrarian; 2 females, 1 male, litter in oak forest, Zagros Mountains, near Patogh ghaut (34°25'N, 46°00'E / 1030 m a.s.l.), Sarpol-e-zahab County, Kermanshah Province, Iran, 9.II.2014, leg. M. Kahrarian; 1 male, oak forest, Zagros Mountains, near Shabankareh village (34°52'N, 46°30'E / 1600 m a.s.l.), Paveh County, Kermanshah Province, Iran, 20.I.2014, leg. M. Kahrarian. Holotype and 7 paratypes deposited at the Department of Agronomy, Kermanshah Branch, Islamic Azad University, Kermanshah, Iran and 4 paratypes deposited in the collection of the Institute of Environmental Biology, University of Wrocław, Poland.

**Figures 1–3.**
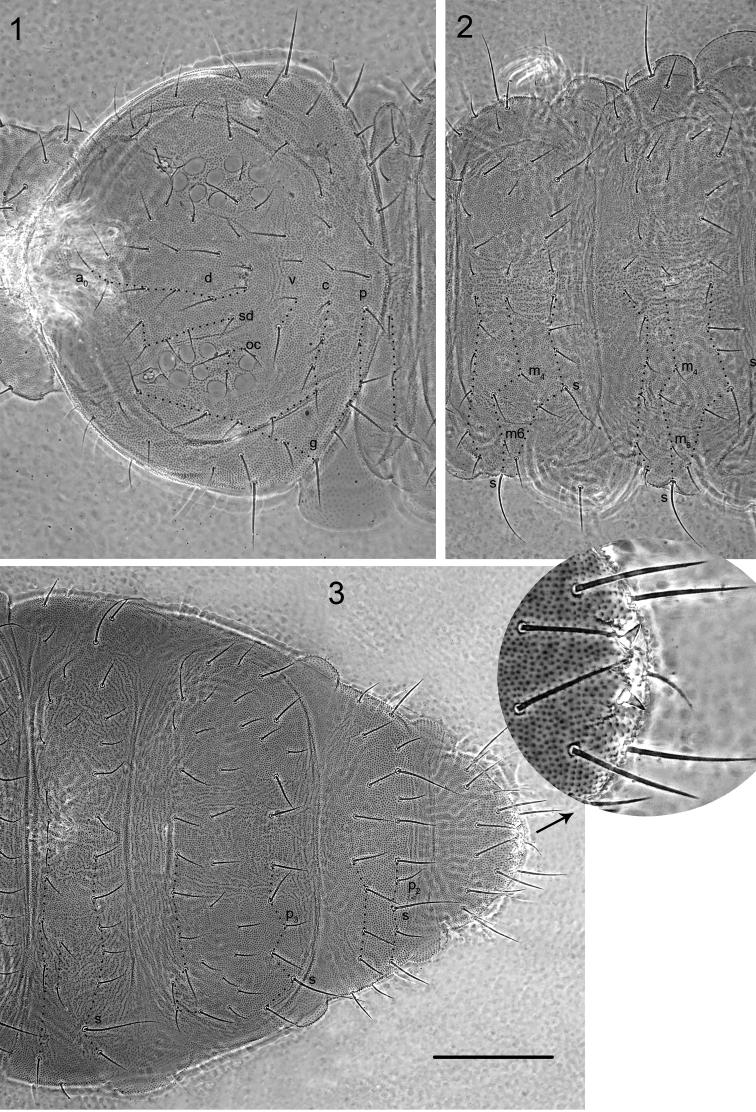
*Hypogastrura ellisi* sp. n. **1** chaetotaxy of head and Th. I **2** chaetotaxy of Th. II–III **3** chaetotaxy of Abd. III–VI. Scale bar: 0.1 mm.

#### Other material.

Greece, Lesbos, leg. Ellis (deposited at the Naturalis Biodiversity Center, Netherland): 22 females, 6 males (21038–21054, 21056–21059, 21062–21064, 21066–21068), Antissa, 30.X.1973, 973240; 18 females, 23 males (21060, 21078–21117), Antissa, 30.X.1973, 973243; 1 male (21061), Antissa, 30.X.1973, 973244; 1 male (21037), Gavathas, 31.X.1973, 973247.

#### Etymology.

Dedicated to Dr. Willem N. Ellis, an excellent specialist in Collembola.

#### Diagnosis.

Habitus typical of genus. Ant. IV with three lateral and one dorsal long thin and curved blunt sensilla (sometimes 1–2 more in dorsal group, longer and less curved). Postantennal organ equal to, or slightly larger than, nearest ocellus. Labrum with distinct apical papillae. Tibiotarsi with one clavate tenent hair. Empodial lamellae broad. Ventral tube with 4 + 4 setae. Retinaculum with 4 + 4 teeth. Dens with fine, uniform granulation and seven setae. Mucro with comparatively high outer lamella. Anal spines small, situated on low basal papillae.

#### Description.

Body length 1.1–1.6 mm. Habitus typical of the genus. Color in alcohol pale brown dorsally and yellowish ventrally, eye-patches dark. Granulation fine and uniform, 12–20 granules between setae p_1_ on Abd. V.

Chaetotaxy of head typical of the genus, with complete set of v-setae (Fig. 1). Setae slightly differentiated in length, especially on last abdominal segments, smooth and rather thick and stiff. Body sensilla (s) about 2–3 times longer than ordinary setae, fine and smooth. Dorsal chaetotaxy of Th. I–III and Abd. III–VI as in Figs 1–3. Th. I with 3 + 3 setae. Th. II with setae m_2_ absent, m_3_ present or absent and m_4,_ m_6_ present. Th. III with setae m_2_ and m_3_ absent and setae m_4_ and m_6_ present. Abd. IV with setae p_3_ present, p_7_ absent and increased number of m-setae. On Abd. V setae p_2_ present and m-setae absent. Subcoxae I, II, III with 1, 3, 3 setae respectively. Microsensillum on Th. II present.

**Figures 4–8.**
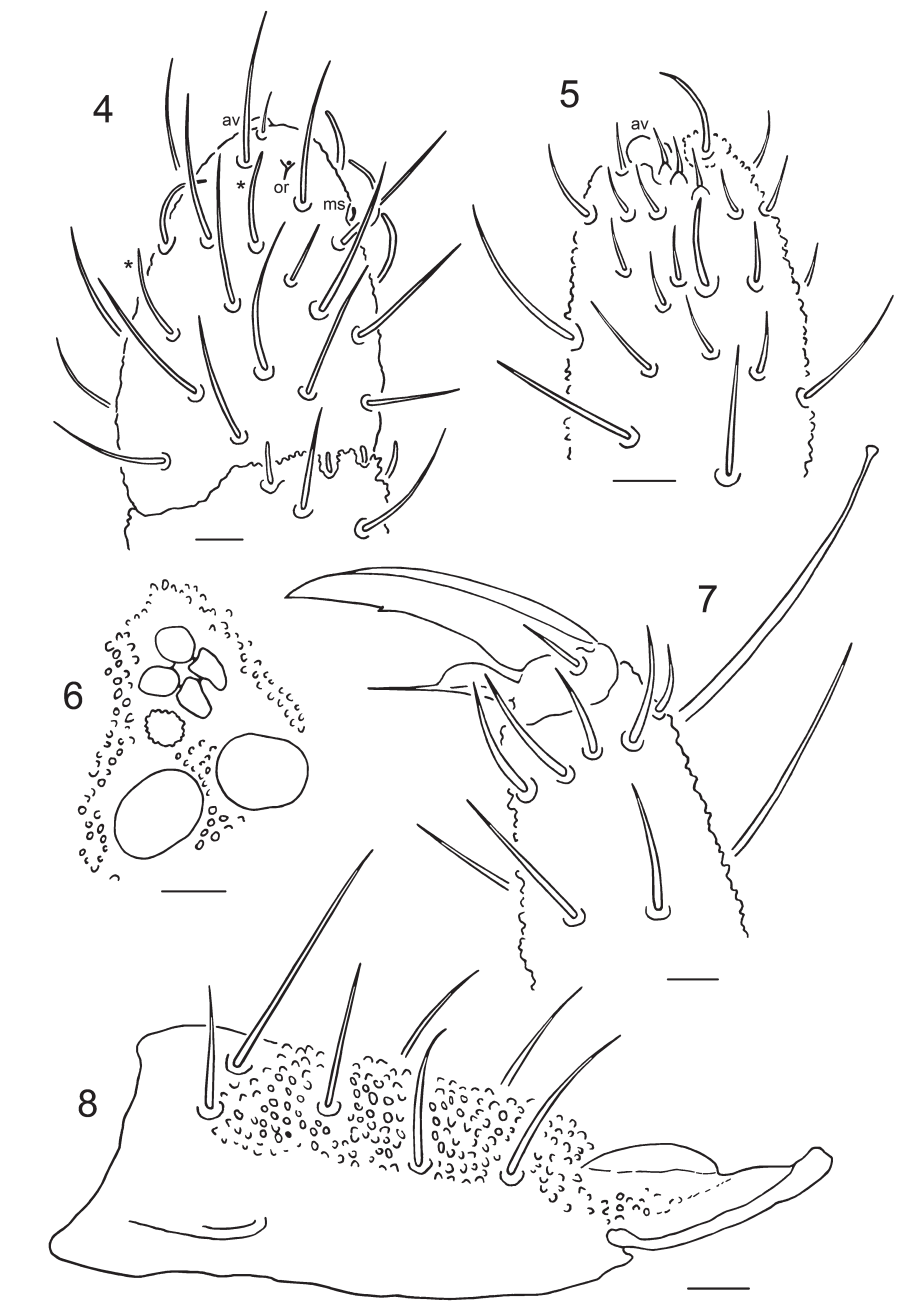
*Hypogastrura ellisi* sp. n. **4** chaetotaxy of dorsal side of Ant. IV **5** chaetotaxy of ventral side of Ant. IV **6** postantennal organ and neighbor ocelli **7** claw III **8** dens and mucro. Abbreviations in text. Scale bars: 0.01 mm.

Ant. IV with simple apical vesicle, subapical organite (or), microsensillum (ms), three lateral and one dorsal long thin and curved blunt sensilla (sometimes 1–2 more in dorsal group, longer and less curved, marked with an asterisk in Fig. 4) and 5–10 short pointed setae in ventral file (Fig. 5). Ant. III-organ with two long (outer) and two short (inner) sensilla (Fig. 4). Microsensillum on Ant. III present. Ant. I with seven setae (seta p’ absent).

**Figures 9–14.**
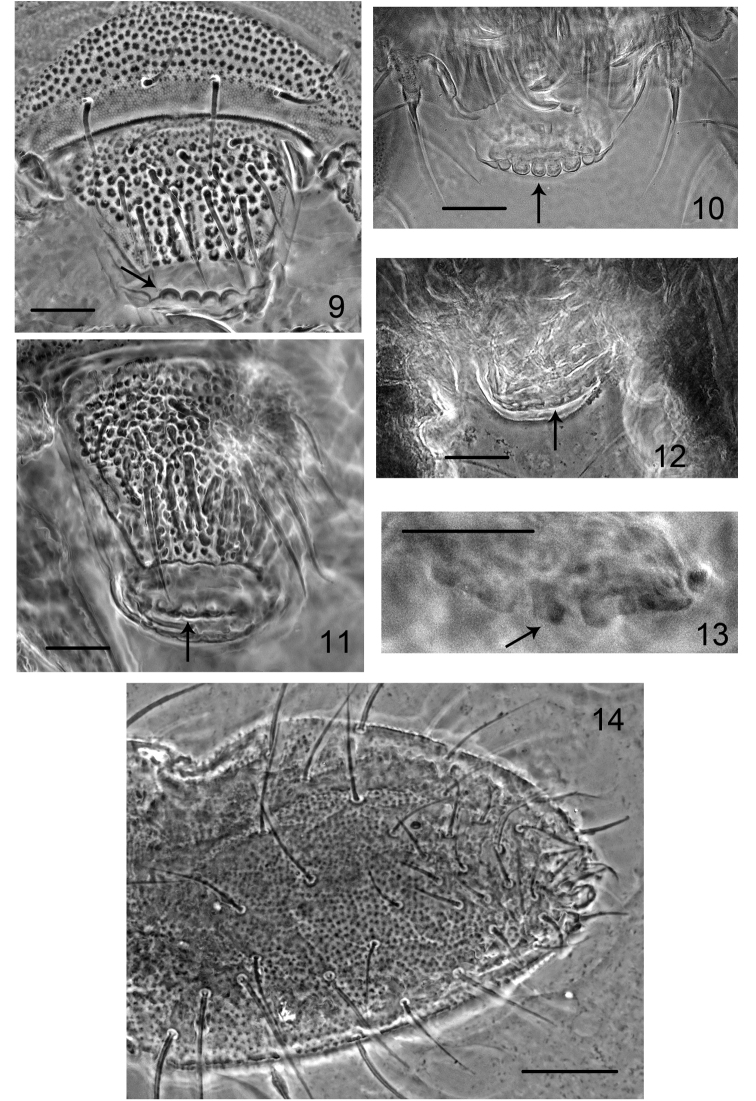
*Hypogastrura ellisi* sp. n. **9** labrum **10** apical part of labrum, *H. tethyca*: **11** labrum, specimen from Azerbaijan **12** apical part of labrum, specimen from Crete **13** apical papillae, specimen from Crete **14** chaetotaxy of ventral side of Ant. IV. Black arrows indicate apical papillae. Scale bars: 0.01 mm.

Ocelli 8 + 8. Postantennal organ equal to, or slightly larger than, neighboring ocellus, with four subequal lobes. Accessory boss present (Fig. 6). Labrum with six distinct apical papillae (four large and two smaller, Figs 9–10). Labral setae 5, 5, 4, prelabrals 4. Maxillary head of the *H. tullbergi* type (Fjellberg 1984) and labium as in Fjellberg (1999: fig. 6). Outer lobe of maxilla with two sublobal hairs.

Tibiotarsi I, II, III with 19, 19, 18 setae respectively. Apical seta A_1_ long and clavate. Claws with small inner tooth. Empodial appendage with broad basal lamella and apical filament reaching slightly beyond inner tooth of unguis (Fig. 7).

Ventral tube with four setae on each side. Retinaculum with 4 + 4 teeth.

Furca well developed (ratio dens + mucro/inner edge of claws III 2.4–2.7). Dorsal side of dens with fine, uniform granulation and seven setae. Mucro with relatively high outer lamella. Ratio dens/mucro 2.0–2.3 (Fig. 8).

Anal spines small, situated on low basal papillae (Fig. 3), ratio anal spine/basal papilla 0.7–1.1, ratio anal spine + basal papilla/inner edge of claws III 0.5–0.6.

#### Remarks.

It is difficult to find a right place for *H. ellisi* sp. n. within the genus. This species does not key to any of the groups in Skarżyński (2009). However, having weakly differentiated blunt Ant. IV sensilla, one tenent hair on the tibiotarsi, broad empodial lamellae, dens without tooth-like granules and ventro-apical swelling and a mucro without a distinct subapical tooth, it can be compared with some representatives of the *trybomi* or *monticola* groups as well as *H. aterrima* Yosii, 1972, which has an isolated position within the genus.

Undoubtedly, *H. ellisi* sp. n. is the most similar to *H. tethyca*, considered as a member of the *trybomi* group. Most noticeably they differ in the shape of labral apical papillae (*H. ellisi* sp. n. – convex, strong, well visible, Figs 9–10; *H. tethyca* – flat, delicate, hardly visible, Figs 11–13). Apart from this *H. ellisi* sp. n. lacks setae m_2_ on Th. II (present in *H. tethyca*) and possesses smaller anal spines (the ratio of anal spine + basal papilla/inner edge of claws III 0.5–0.6 in *H. ellisi* sp. n. vs 0.75–1.1 in *H. tethyca*), 5–10 short pointed setae in the ventral file on Ant. IV (Fig. 5) (*H. tethyca* – approx. ten short and stiff sensilla, truncate at apex, Fig. 14), and a mucro with a relatively high outer lamella (both inner and outer lamellae are low in *H. tethyca*).

Both species have similar general ranges of distribution (*H. ellisi* sp. n.: Iran, Greece – Lesbos; *H. tethyca*: Greece – Crete, Lesbos and Azerbaijan), but on a local scale they co-occur only in Lesbos. In Ellis’s material from this island, numerous *H. ellisi* sp. n. from two localities and rare *H. tethyca* individuals from five sites were found. Nevertheless, they were isolated spatially. Unfortunately, due to incomplete collecting data, we do not know whether these populations differ in habitat preferences.

The new species is easy to distinguish from the members of the *monticola* group by the absence of m-setae on Abd. V (vs present) and the size of the postantennal organ, which is equal to, or slightly larger than, the neighboring ocellus (vs 1.5–2 times larger than ocellus). *H. aterrima* can also be easily separated from *H. ellisi* sp. n. due to tridentate retinaculum (vs quadridentate), minute anal spines, slightly larger than surrounding granules (vs large, the ratio of anal spine + basal papilla/inner edge of claws III 0.5–0.6 in *H. ellisi* sp. n.), the presence of setae m_2_ on Th. II, and the absence of setae m_6_ on Th. II–III.

### Hypogastrura tethyca

Taxon classificationAnimaliaCollembolaHypogastruridae

Ellis, 1976

Figs 11–14

#### Type material.

Paratypes: Greece, Crete, leg. A.C. & W.N. Ellis (deposited at the Naturalis Biodiversity Center, Netherland): 2 females (21008, 21010), 2 males (21011, 21013), Knossos, loose loam, sparsely grown with grass and *Oxalis pes-caprae* L. at foot of a 4-m high cliff along road, 24.X.1972, 972.219; 3 females (21017, 21018, 21036), 3 males (21028, 21029, 21030), Knossos, collected manually under stones pieces of dead wood etc. 24.X.1972, 972.195; 1 female (21004), Iraklion, 25.X.1972, after heavy rains, loam, litter and rotting leaf bases under vigorous ruderal vegetation (*Ecballium elaterium* (L.) Rich., 972.233; 1 male (21015), Marathos, 15 km W of Iraklion, 26.X.1972, litter under *Pistacia lentiscus* in phrygana on weak north slope, 972.209; 3 females (20978, 20979, 20980), 7 males (20983, 20984, 20985, 20986, 20987, 20988, 20989), Malia, litter of *Quercus coccifera* in well-developed phrygana, 29.X.1972, 972.211.

#### Other material.

Greece, Lesbos, leg. Ellis (deposited at the Naturalis Biodiversity Center, Netherland): 2 females (21069), 1 male (21070), Alifanta, 24.X.1973, 973219; 1 female (21075), Ayiásos, 16.XI.1973, 973334; 2 females (21072, 21073), 1 male (21074), Profitis Ilias, 13.X.1973, 973312; 1 female (21077), 1 male (21076), Agia Marina, 23.XI.1973, 973367; 1 female (21055), Mitilini, 19.X.1973, 973102. Azerbaijan, leg. Z.K.Rasulova (deposited at the Severtsov Institute of Ecology & Evolution, Russian Academy of Sciences, Moscow): 7 females, 3 males (other data unknown).

#### Note.

The description (Ellis 1976) and redescription (Babenko et al. 1994) of *H. tethyca* are highly informative; however, the examination of the types and other material allow us to add some more details. *Hypogastrura tethyca* has 10–18 granules between setae p_1_ on Abd. V, Ant. IV with three lateral and one dorsal long thin and curved blunt sensilla (sometimes 1–2 more in the dorsal group, longer and less curved, in arrangement as in *H. ellisi* sp. n., Fig. 4) and approximately ten short stiff sensilla truncate at the apex in the ventral file (Fig. 14), the postantennal organ 1.0–1.5 (usually 1.2) as long as the nearest ocellus, the labrum with delicate flat and hardly visible apical papilla (Figs 11–13), a maxillary outer lobe with two sublobal hairs, and large anal spines on high papillae (the ratio anal spine + basal papilla/inner edge of claws III 0.75–1.1).

Ellis (1976), looking for *H. tethyca* affinities, pointed out difficulties with its placement within the genus and compared it with a wide spectrum of species, including *H. monticola* Stach, 1946, *H. aterrima*, and also *H. trybomi* (Schött, 1893). Then, Babenko et al. (1994), based on specimens from Azerbaijan, put this species into the *trybomi* group. Although this concept seems well justified, *H. tethyca*, having a labrum with delicate apical papillae and tibiotarsi with long and clavate tenent hair, occupies a rather isolated position within the group. Undoubtedly, further research is needed to establish its relationships. The characteristics of the *trybomi* group and a key to the known species of the group are given below. *H. tethyca* is also similar to *H. ellisi* sp. n. They differ in the characters mentioned above.

### Notes on the *trybomi* group

The *trybomi* group was created by Christiansen and Bellinger (1980) for five Nearctic species: *H. irenae* (Wray, 1953), *H. lima* Christiansen & Bellinger, 1980, *H. maynardi* Christiansen & Bellinger, 1980, *H. oregonensis* Yosii, 1960, and *H. trybomi*. Interestingly, subsequent studies (Fjellberg 1985, Babenko et al. 1994, Babenko and Fjellberg 2006) showed that *H. trybomi* sensu Christiansen and Bellinger (1980) rather referred to *H. oregonensis*. Afterwards, Babenko et al. (1994) supplemented the Christiansen and Bellinger (1980) definition with new essential features and enlarged the group by adding species recorded in Palearctic: *H. maxillosa* Babenko, 1994 and *H. tethyca*.

Presently, after the recent description of some new species (Skarżyński 2007, Jiang and Yin 2010, 2012, Jia et al. 2011) and the redescription of some poorly defined ones (Bernard 2015), twelve species can be included into the *trybomi* group sensu Christiansen and Bellinger (1980, 1998) and Babenko et al. (1994): *H. analpapillata* Jiang & Yin, 2012, *H. hargrovei* Skarżyński, 2007, *H. gravesi* Wray, 1971, *H. heptasetata* Jiang & Yin, 2010, *H. hexasetata* Jiang & Yin 2010, *H. irenae*, *H. lima*, *H. manghe* Jia, Skarżyński & Konikiewicz, 2011, *H. maxillosa*, *H. oregonensis*, *H. tethyca*, and *H. trybomi*. Another one, *H. maynardi*, can also be considered a potential member of this group. However, a modern redescription of this species is necessary to solve this problem (Christiansen and Bellinger 1998).

These species have fine cuticular granulation of the body (7–18 cuticular granules between setae p_1_ on Abd. V), long and thin blunt Ant. IV sensilla arranged in two groups: 2–3 lateral and 1–8 dorsal (often difficult to distinguish from ordinary setae), a labrum without distinct apical papillae, a postantennal organ from slightly smaller to slightly larger than the neighboring ocellus, one usually short pointed tenent hair on the tibiotarsi (only in *H. tethyca* clavate), a broad basal empodial lamella, a quadridentate retinaculum, dens with 6–7 setae and without tooth–like granules and ventro–apical swelling, a mucro without distinct subapical tooth, setae m_6_ on Th. II–III present, m–setae on Abd. V absent, and usually 4 + 4 setae on the ventral tube (only in *H. trybomi* 7–9 + 7–9). Moreover, some of them have the head of the maxilla with prolonged lamellae, a maxillary outer lobe with only one sublobal hair, and Ant. IV with a developed ventral file of sensilla. Members of this group differ in the characters summarized in Table 1 and a key.

**Table 1.** Morphological differences between the members of the *trybomi* group. Data after: *H. analpapillata* – Jiang and Yin (2012); *H. hargrovei* – Skarżyński (2007); *H. gravesi* – Bernard (2015); *H. heptasetata* – Jiang and Yin (2010); *H. hexasetata* – Jiang and Yin (2010); *H. irenae* – Bernard (2015); *H. lima* – Christiansen and Bellinger (1998), Skarżyński (2007); *H. manghe* – Jia et al. (2011); *H. maxillosa* – Babenko et al. (1994); *H. oregonensis* – Yosii (1960), Fjellberg (1985), Babenko et al. (1994), Christiansen and Bellinger (1998), Skarżyński (2007); *H. tethyca* – Ellis (1976), Babenko et al. (1994) and own data; *H. trybomi* – Fjellberg (1985), Babenko et al. (1994). Abbreviations: blAnt – number of blunt sensilla on Ant. IV, venAnt – number/shape of sensilla in ventral file on Ant. IV (tips: p – pointed, t – truncate, b – broadened and flattened), lam – prolonged maxillary lamellae 4 and 5, sl – number of sublobal hairs in maxillary outer lobe, labC – papilla C in labial palp, vhead – number of axial setae on ventral side of head, m2 – setae m_2_ on Th. II., setD – number of setae on dens, granD – coarse cuticular granulation on dens (at least in distal part), As/pap – ratio anal spine/basal papilla.

**Species**	**blAnt**	**venAnt**	**lam**	**sl**	**labC**	**vhead**	**m2**	**setD**	**granD**	**As/pap**
*H. analpapillata* ^1^	7	35–50/p	+	2	+	3 + 3	+	7	+?	0.4
*H. gravesi* ^2^	4–5	40–50/b	-	2	+	?	+	7	+	1.5–2
*H. hargrovei* ^3^	6	ca. 10/p	+	1	-	2 + 2	-	6	-	ca. 1
*H. heptasetata* ^4^	10	10-15/p	+	1	-	2 + 2	+	7	-	1.8
*H. hexasetata*	8–10	30–55/p	+	1	+	3 + 3	+	6	-	ca. 1
*H. irenae*	8	53–58/b ^5^	-	1	+	?	+	7	+	ca. 1
*H. lima*	7–8	ca. 20/b	-	?	?	?	?	7	+	1.5–2
*H. manghe* ^6^	9–11	30–45/b	+	1	+	3 + 3	+	6	-	ca. 1
*H. maxillosa*	7–9	ca. 20/? ^7^	+	2	?	3 + 3	-	7	-	ca. 1
*H. oregonensis*	7–9	20–35/b ^8^	-	2	+	3 + 3	+/-	6–7	+	ca. 1
*H. tethyca* ^9^	4–6	ca. 10/t	-	2	+	3 + 3	+	7	-	ca. 1
*H. trybomi* ^10^	?	ca. 10/p	-	2	?	3 + 3	-	7	-	ca. 1

^1^ Basal papillae of anal spines strongly granulated and fused to each other

^2^ Anal spines blunt, rounded or truncated apically

^3^ Labrum elongated, head of maxilla with two teeth, hypostomal setae of labial palp set on a narrow long projection

^4^ Maxillary lamellae 6 longer than teeth

^5^ Sensilla thick

^6^ Ant. IV with trilobed apical vesicle

^7^ After Babenko et al. (1994: fig. 20.6)

^8^ After Fjellberg (1985: fig. 86) and Babenko et al. (1994: fig. 19.2)

^9^ Labrum with flat delicate hardly visible apical papillae, tibiotarsi with clavate tenent hair

^10^ Ventral tube with 7–9 + 7–9 setae, tibiotarsi with relatively long, but pointed tenent hair

Considering their morphology, one can conclude that three species: *H. tethyca*, *H. trybomi*, and *H. hargrovei*, occupy rather isolated positions. Especially the first one due to the reasons mentioned above, the second one because of the ventral tube with numerous setae and tibiotarsi with relatively long, but pointed, tenent hair (judging from fig. 111 in Fjellberg (1985) and fig. 17.5 in Babenko et al. (1994)), and the third one due to highly modified mouthparts: the labrum elongated, the head of the maxilla with only two teeth and prolonged lamellae, the labial palp without papilla C, with hypostomal setae set on a narrow long projection, and a weakly developed ventral file of Ant. IV sensilla.

The remaining nine species form two subgroups: Eastern Palearctic (*H. analpapillata*, *H. heptasetata*, *H. hexasetata*, *H. manghe*, and *H. maxillosa*), with distinctly prolonged maxillary lamellae (lamellae 1, 2, 4, 5 exceed maxillary teeth) and fine cuticular granulation on dens, and Nearctic (*H. gravesi*, *H. irenae*, *H. lima*, and *H. oregonensis*), characterized by maxillary lamellae longer than in *H. tethyca* or *H. trybomi* (maxilla of the *tullbergi* type) but distinctly shorter than in representatives of the previous subgroup (at most lamellae 1 and 2 exceed maxillary teeth) and dens (at least in distal part) with coarse cuticular granulation.

The general distribution of the group is Holarctic; however, only one member, *H. oregonensis*, lives in both Palearctic and Nearctic (W Nearctic – USA: Alaska, California, Idaho, Montana, Oregon, Washington; Canada: Northwest Territories; E Palearctic – Russia: Chukotka; Japan) (Hammer 1953, Yosii 1960, Fjellberg 1985, Babenko et al. 1994, Christiansen and Bellinger 1998, Babenko and Fjellberg 2006). The remaining species have more restricted geographic ranges. *H. tethyca* occurs in Greece (Crete, Lesbos) and Azerbaijan; *H. trybomi* lives in high Arctic regions of Palearctic (from Franz Josef Land to Wrangel Island, Babenko and Fjellberg 2006); *H. maxillosa* is known from one location in Middle Siberia (Tomskaya oblast) (Babenko et al. 1994). Four species occur in China: *H. analpapillata* (Yunnan Province) (Jiang and Yin 2012), *H. heptasetata* (Jiangsu Province) (Jiang and Yin 2010), *H. hexasetata* (Hubei Province) (Jiang and Yin 2010), and *H. manghe* (Shanxi Province) (Jia et al. 2011), and four in the eastern states of the USA: *H. gravesi* (North Carolina) (Bernard 2015), *H. hargrovei* (South Carolina) (Skarżyński 2007), *H. irenae* (North Carolina) (Bernard 2015), and *H. lima* (Pennsylvania, Maryland, New York) (Christiansen and Bellinger 1998).

### Key to *Hypogastrura* species of the *trybomi* group

**Table:** 

1	Tenent hair on tibiotarsi pointed	**2**
–	Tenent hair on tibiotarsi clavate	***H. tethyca* Ellis, 1976**
2	Ventral tube with 4 + 4 setae	**3**
–	Ventral tube with 7–9 + 7–9 setae	***H. trybomi* (Schött, 1893)**
3	Labrum short	**4**
–	Labrum elongated	***H. hargrovei* Skarżyński, 2007**
4	Maxillary lamellae 4 and 5 equal to or shorter than teeth	**5**
–	Maxillary head with lamellae 4 and 5 much longer than teeth	**8**
5	Ant. IV with 7–9 blunt sensilla, anal spines conical	**6**
–	Ant. IV with 4–5 blunt sensilla, anal spines blunt, rounded or truncated apically	***H. gravesi* Wray, 1971**
6	Anal spines shorter than or subequal to papillae	**7**
–	Anal spines 1.5–2 times as long as papillae	***H. lima* Ch & B, 1980**
7	Maxillary outer lobe with one sublobal hair	***H. irenae* (Wray, 1953)**
–	Maxillary outer lobe with two sublobal hairs	***H. oregonensis* Yosii, 1960**
8	Th. II with setae m_2_ present	**9**
–	Th. II with setae m_2_ absent	***H. maxillosa* Babenko, 1994** (in Babenko et al. 1994)
9	Seven setae on dens	**10**
–	Six setae on dens	**11**
10	Ant. IV with 10–15 sensilla in ventral file, head ventrally with 2 + 2 axial setae, labial palp with papilla C absent, maxillary outer lobe with one sublobal hair, ratio anal spine : basal papilla 1.8	***H. heptasetata* Jiang & Yin 2010**
–	Ant. IV with 35–50 sensilla in ventral file, head ventrally with 3 + 3 axial setae, labial palp with papilla C present, maxillary outer lobe with two sublobal hairs, ratio anal spine : basal papilla 0.4	***H. analpapillata* Jiang & Yin, 2012**
11	Ant. IV with simple apical vesicle and sensilla in ventral file pointed	***H. hexasetata* Jiang & Yin 2010**
–	Ant. IV with trilobed apical vesicle and sensilla in ventral file broadened and flattened at tips	***H. manghe* Jia, Skarżyński & Konikiewicz, 2011**

## Supplementary Material

XML Treatment for Hypogastrura ellisi

XML Treatment for Hypogastrura tethyca
